# Prognostic significance of LAT1 expression in pleural mesothelioma

**DOI:** 10.1016/j.heliyon.2024.e37414

**Published:** 2024-09-03

**Authors:** Ryo Taguchi, Kyoichi Kaira, Yu Miura, Tetsuya Umesaki, Atsuto Mouri, Hisao Imai, Hiroshi Kagamu, Masanori Yasuda, Yoshikatsu Kanai, Hiroyuki Nitanda, Hironori Ishida, Hirozo Sakaguchi

**Affiliations:** a Department of Thoracic Surgery, Japan; b Department of Respiratory Medicine, Japan; c Department of Pathology, Saitama Medical University, International Medical Center, 1397-1, Yamane, Hidaka City, 350-1298, Japan; d Department of Bio-system Pharmacology, Graduate School of Medicine, Japan; e Premium Research Institute for Human Metaverse Medicine (WPI-PRIMe), Japan; f Integrated Frontier Research for Medical Science Division, Institute for Open and Transdisciplinary Research Initiatives (OTRI), Osaka University, Osaka, Japan

**Keywords:** Malignant mesothelioma, LAT1, Prognosis, Immunohistochemistry, Predictive marker

## Abstract

**Background:**

The L-type amino acid transporter (LAT1) exhibits significantly increased expression within tumor cells across various neoplasms. However, the clinical significance of LAT1 expression in patients with pleural mesothelioma (PM) remains unclear.

**Methods:**

Eighty patients diagnosed with PM between June 2007 and August 2022, were eligible for this study. LAT1, alanine-serine-cysteine transporter 2 (ASCT2), Ki-67, and VEGFR2 were evaluated by immunohistochemistry. Inflammatory and nutritional indices were also correlated with different variables, including neutrophil to lymphocyte ratio (NLR), platelet to lymphocyte ratio (PLR), systemic immune-inflammation index (SII), prognostic nutritional index (PNI), advanced lung cancer inflammation index (ALI), and Glasgow prognostic score (GPS).

**Results:**

LAT1 was highly expressed in 57.5 % of patients with PM. Among the 80 patients included in this study, 65 (81.3 %) received chemotherapy, either alone or followed by surgical resection, while 15 (18.7 %) opted for best supportive care. The level of LAT1 significantly correlated with cell proliferation and ASCT2. Factors such as performance status, histology, LAT1 expression, PNI, ALI, and GPS were significant prognostic indicators for progression-free survival (PFS), while Ki-67, LAT1, NLR, SII, PNI, ALI, and GPS were identified as significant predictors for overall survival (OS). LAT1 expression emerged as an independent prognostic factor for predicting PFS and OS in all patients, as well as in the subgroup of 65 patients receiving chemotherapy. Notably, high LAT1 expression proved to be a significant predictor of outcome, particularly in the subgroup with high PLR and SII.

**Conclusion:**

LAT1 was a significant predictor of outcomes in patients with PM and was more predictive of worse outcomes in patients with high inflammatory and low nutritional status.

## 1 Introduction

Amino acids play a crucial role in the progression of many cancer cells, while amino acid transporters are essential for growth and proliferation in normal and transformed cells [[1], [2], [3]]. Among amino acid transporters, system L is a Na^+^–independent large and neutral amino acid transport agency [2]. L-type amino acid transporter 1 (LAT1) is part of system L amino acid transporter subtype (LAT1, LAT2, LAT3, and LAT4) and is closely associated with tumor cell proliferation, angiogenesis, and survival across various human neoplasms [[1], [2], [3], [4]]. LAT1 transports large neutral amino acids such as leucine, isoleucine, valine, phenylalanine, tyrosine, tryptophan, methionine, and histidine, and requires covalent association with the heavy chain of 4F2 cell surface antigen (4F2hc) for its functional expression on plasma membrane [1,2]. Full-length of LAT1 was firstly isolated and characterized in 1998 [1]. LAT1 is widely expressed in human neoplasms and supplies the essential amino acids to enhance the growth of cancer cells via mammalian target-of-rapamycin (mTOR) stimulated translation [3]. Although overexpression of LAT1 is known to be linked to metastases and angiogenesis, the inhibition of LAT1 function could be a possible therapeutic agent for many types of cancer [[3], [4], [5]]. Several previous studies identified LAT1 as a prognostic marker in several human cancers, but not enough evidence for LAT1 expression as predictive marker after any treatment [3,4].

Pleural mesothelioma (PM) is uncommon malignant neoplasms arising from mesothelial cells including pleura, pericardium, and peritoneum, and is an aggressive neoplasm characterized by its resistance to various treatment modalities. PM is categorized into three primary histological types; epithelioid, sarcomatoid, and biphasic types. Some of the patients received the comprehensive treatment including a combination of systemic chemotherapy, radiotherapy, and surgical resection, however, most patients do not receive surgery or radiotherapy. Based on recent evidence, immunotherapy, such as nivolumab or nivolumab plus ipilimumab has been identified as one of the standard option for the treatment of PM [6,7]. However, there remains a lack of established biomarkers for predicting treatment outcomes in patients with PM. Further investigation is warranted to discover an established biomarker after any treatment in the patients with PM.

We previously investigated the relationship between LAT1 expression and other biomarkers in 21 patients with PM [8]. Our preliminary data demonstrated that LAT1 is highly expressed and closely correlated with hypoxia, mTOR pathway, and tumor progression [8]. However, the small sample size may introduce bias about the prognostic potential of LAT1 expression in the patients with PM. In addition, amino acid metabolism, such as LAT1, is biologically linked to the inflammatory and nutritional status in the tumor environment [9]. Recent studies have reported that inflammatory and nutritional indices such as the neutrophil-to-lymphocyte ratio (NLR), platelet-to-lymphocyte ratio (PLR), systemic immune-inflammation index (SII), advanced lung cancer inflammation index (ALI), prognostic nutritional index (PNI), and Glasgow prognostic score (GPS) could closely correlate with worse outcomes after immunotherapy in patients with lung cancer [[10], [11], [12], [13]]. However, whether LAT1 expression differs according to these inflammatory and nutritional indices remains unclear in the patients with PM. Another amino acid transporter, the alanine-serine-cysteine transporter 2 (ASCT2), is highly expressed in human neoplasm [3,14]. ASCT2 expression closely correlated with LAT1 expression and poor survival in patients with advanced lung cancer [14]. In addition, a significant correlation between ASCT2 expression and cell proliferation by Ki-67 and vascular endothelial growth factor (VEGF) has been observed in lung cancer. Although the tumor immune microenvironment, including tumor-infiltrating lymphocytes (TILs), has been identified as a target for immunotherapy, the relationship between amino acid transporters and TILs remains unclear. In particular, little is known about the prognostic significance of LAT1 expression in relation to ASCT2, angiogenesis, TILs, and inflammatory or nutritional parameters.

Given these considerations, we conducted a clinicopathological study to examine the prognostic significance of LAT1 expression in patients with PM. Our study aimed to correlate LAT1 expression with inflammatory and nutritional markers, angiogenesis, tumor progression, TILs, and other amino acid transporters.

## 2 Methods

### 2.1 Patients

Eight-five patients were histologically confirmed to have PM at our institution between June 2007 and August 2022. Among them, five patients did not have sufficient tumor specimens for immunohistochemistry before receiving any treatment. Therefore, 80 patients were finally enrolled in this study. Clinical data were extracted from the medical records. This study was approved by the Institutional Ethics Committee of the International Medical Center of Saitama Medical University (approval no. 2024-15), Hidaka City, Japan. Owing to the retrospective nature of the study, the Ethical Committee waived the requirement for obtaining written informed consent from the patients for the use of human tissues in the study [15].

### 2.2 Immunohistochemistry

Immunohistochemical staining was performed as previously described [[16], [17], [18]]. LAT1 expression was determined via immunohistochemistry by incubating tumor specimens with a mouse monoclonal antibody against LAT1 [16] at a dilution of 1:5000 in phosphate-buffered saline containing 0.1 % bovine serum at 4 °C overnight. This was followed by a subsequent incubation at room temperature for 30 min. LAT1 expression was considered positive only when distinct membrane staining was observed. The percentage of LAT1 staining was scored as follows: 1, 0–10 %; 2, 11–25 %; 3, 26–50 %; and 4, 51–100 %. Staining intensity was not considered when assessing the staining outcomes. Expression was defined as “high” when tumors contained cancer cells that were assigned staining scores of 3 or 4. Ki-67 mouse monoclonal antibody (MIB-1, 1:100 DAKO, Glostrup, Denmark; M7240), ASCT2 rabbit monoclonal antibody (D7C12, 1:100 Cell Signaling Technology, Inc., #8057), mouse monoclonal CD8 (DAKO, clone C8/144B 1/150 high pH retrieval), and mouse monoclonal FOXP3 (Abcam, 1/50, clone 236A/E7, pH6 retrieval) were quantified based on previous procedures [[17], [18], [19]]. The cutoff values of Ki-67, CD8, FOXP3, and ASCT2 were the median values, and high and low expression levels were defined as the median values for each marker. VEGFR2 rabbit polyclonal antibody (1:100; Cell Signaling Technology, Inc.; cat #2472) was scored based on the stained tumor area as follows: 1, ≤10; 2, 11–24; 3, 25–49; and 4, ≥50 % staining. Low and high expression levels were defined as scores of 1–3 and 4 for VEGFR2, respectively, as previously described [17,18].

The sections were evaluated by at least two researchers (RT and KK) using a light microscope ( × 200 and × 400 magnification) in a blinded manner. In the case of discrepancies, both investigators simultaneously evaluated the slides until a final consensus was reached. The investigators were blinded to patient outcomes.

### 2.3 Assessment of the inflammatory and nutritional indices

Clinical and biological data [e.g., total protein, albumin, and C-reactive protein (CRP) levels; white blood cell, neutrophil, platelet, and lymphocyte counts; and height and weight] were extracted from medical records for analysis. Six indices reflecting systemic inflammatory and nutritional status based on previous studies [10] were calculated at baseline within 1 week of the first cycle of each treatment. The inflammatory indices used were NLR [11], PLR [11], and SII (platelet count × neutrophil count/lymphocyte count) [11]. The nutritional indices included were PNI = 10 × albumin (g/dL) + 0.005 × lymphocyte count [10], ALI = BMI + albumin level (g/dL)/NLR [12], and GPS. The GPS was tabulated as follows: 0 = no abnormal values (good), 1 = one abnormal value (intermediate), and 3 = two abnormal values (poor) [13]. Abnormal values included CRP >10 mg/mL and albumin <3.5 g/dL. A GPS of 0 was defined as low, while a GPS of 1 or 2 was defined as high. The cutoff value for these inflammatory and nutritional markers was defined as the median value, and the expression of these markers was considered as high if more than median value.

### 2.4 Statistical analysis

Student's *t-*test and the χ^2^ test were used for continuous and categorical variables, respectively. The statistical significance level was set at *P* < 0.05. Correlations between different markers and LAT1 expression were analyzed using Spearman's rank test. PFS was defined as the time from initial treatment to disease progression or death. OS was defined as the time from initial treatment to death from any cause. The Kaplan–Meier method was used to estimate survival as a function of time, and survival differences were analyzed using the log-rank test. Univariate and multivariate analyses of different variables were performed using logistic regression analysis. All statistical analyses were performed using GraphPad Prism (v.10.0; GraphPad Software, San Diego, CA, USA) and JMP 16.0 (SAS Institute Inc., Cary, North Carolina, USA).

## 3 Results

### 3.1 Immunohistochemical analysis

**Fig. 1:**
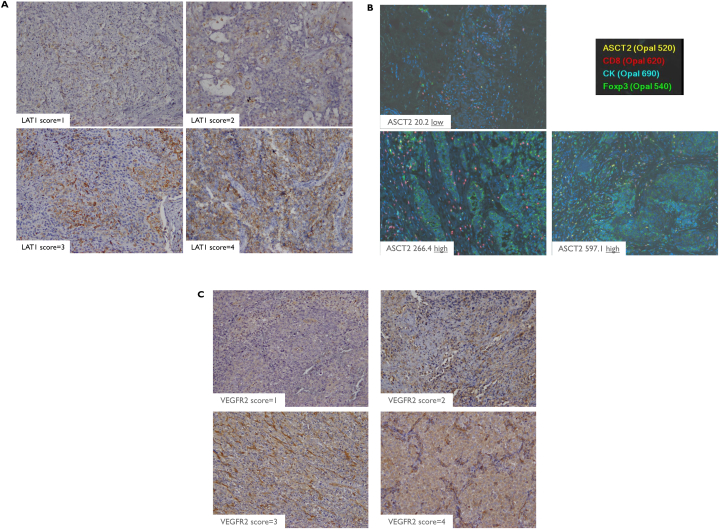
Immunochemistry assay for LAT1, ASCT2, and VEGFR2 in malignant pleural mesothelioma tissue. LAT1 exhibited strong stained on the cell membranes of tumor specimens, with representative images depicting scores of 1, 2, 3, and 4 for LAT1 expression (A). Immunofluorescence via multiplex immunohistochemistry staining demonstrated CD8 (red), Foxp3 (green), and ASCT2 (yellow) (B), showcasing varying levels of ASCT2 expression in tumor specimens. Immunohistochemistry for VEGFR2 expression revealed scores of 1, 2, 3, and 4 (C).

Immunohistochemical staining for LAT1, ASCT2, Ki-67, and VEGFR2 was performed in 80 tumor lesions. Fig. 1 shows representative immunohistochemistry results for LAT1 (Fig. 1A), ASCT2 (Fig. 1B), CD8 (Fig. 1B), FOXP3 (Fig. 1B), and VEGFR2 (Fig. 1C). LAT1 immunostaining was detected in the carcinoma cells of tumor tissues, predominantly localized on the plasma membrane. A high LAT1 expression was observed in 57.5 % (46/80) of cases. The median values of ASCT2, Ki-67, CD8, and FOXP3 were 74.1 (range, 0–597), 6.6 % (0–38.4 %), 8.6 (0–205), and 2.5 (0–53), respectively. High expression levels of ASCT2, Ki-67, CD8, and FOXP3 were observed in 50 % (40/80), 51.3 % (41/80), 52.5 % (42/80), and 52.5 % (42/80) of cases, respectively. VEGFR2 was stained in cytoplasm and nucleus, and the highest VEGFR expression was 42.5 % (34/80).

### 3.2 Patient demographics according to LAT1 expression

Table 1 shows the distribution of different variables according to LAT1 and ASCT2 expression levels in all patients. High Ki-67 levels were significantly associated with LAT1 expression, and smoking history was closely linked to low ASCT2 expression. Of all patients, 20 (25.0 %; 20/80) received systemic treatment followed by surgical resection; 45 (56.3 %; 45/80), systemic treatment without surgical resection; and 15 (18.7 %; 15/80), best supportive care. As a first-line systemic treatment, most (83.1 %; 54/65) patients received platinum-based chemotherapy with pemetrexed; four (6.2 %; 4/65) patients were treated with ipilimumab plus nivolumab, and others (10.7 %; 7/65) received non-platinum-based regimens.

**Table 1:** Patient's demographics according to LAT1 and ASCT2 expression.

Different variables		All pts.	LAT1			ASCT2		
		(n = 80)	High (n = 46)	Low (n = 34)	*p*-value	High (n = 40)	Low (n = 40)	*p*-value
Age	<70/≥70	41/39	26/20	15/19	0.273	17/23	24/16	0.117
Gender	M/F	69/11	40/6	29/5	0.541	33/7	36/4	0.518
ECOG PS	0-1/2-3	64/16	36/10	28/6	0.651	30/10	34/6	0.264
Smoking	Yes/No	60/20	36/10	24/10	0.433	25/15	35/5	**0.011**
Asbestos	Yes/No	33/47	19/27	14/20	0.991	15/25	18/22	0.496
Stage	1-2/3-4	37/43	21/25	16/18	0.901	20/20	17/23	0.501
T factor	1-2/3-4	42/38	24/22	18/16	0.946	24/16	18/22	0.179
N factor	0/1-3	57/23	33/13	24/10	0.911	29/11	28/12	0.805
M factor	0/1	73/7	41/5	32/2	0.359	38/2	35/5	0.432
Histological type	Epi/Non-epi	56/24	30/16	26/8	0.278	29/11	27/13	0.626
Ki-67 LI (%)	H/L	41/39	28/18	13/21	**0.045**	24/16	17/23	0.117
VEGFR2	H/L	34/46	18/28	16/18	0.478	13/27	21/19	0.070
NLR	H/L	39/41	25/21	14/20	0.244	19/21	20/20	0.823
PLR	H/L	39/41	21/25	18/16	0.519	20/20	19/21	0.823
SII	H/L	39/41	23/23	16/18	0.795	18/22	21/19	0.502
PNI	H/L	40/40	20/26	20/14	0.175	18/22	22/18	0.371
ALI	H/L	40/40	21/25	19/15	0.366	19/21	21/19	0.655
GPS	0/1-2	38/42	19/27	19/15	0.197	21/19	17/23	0.371
CD8	H/L	42/38	23/23	19/15	0.655	23/17	19/21	0.502
FOXP3	H/L	42/38	27/19	15/19	0.258	20/20	22/18	0.823

Abbreviations: LAT1, L-type amino acid transporter 1; ASCT2, alanine-serine-cysteine transporter 2; M/F, male/female; H/L, high/low; ECOG, eastern corporative oncology group; PS, performance status; LI, labeling index; epi/non-epi; epithelial type/non-epithelial type; VEGFR2, vascular endothelial growth factor receptor 2; NLR, neutrophil to lymphocyte; PLR, platelet to lymphocyte; SII, systemic immune inflammation; PNI, prognostic nutritional index; ALI, advanced lung cancer inflammation; GPS, Glasgow prognostic score; Pts, patients; Bold font indicates a statistically significant difference.

### 3.3 Correlation of LAT1 with different biomarkers

Correlations between LAT1 and different biomarkers were analyzed using Spearman's rank correlation. The expression level of LAT1 was significantly correlated with Ki-67 and ASCT2 expression but not with other variables (Table 2).

**Table 2:** Correlation of LAT1 with different biomarkers (n = 80).

Different variables	Spearman γ (95 % CI)	*p*-value
Ki-67	0.258 (0.034–0.457)	**0.021**
VEGFR2	0.165 (−0.131 to 0.433)	0.258
ASCT2	0.227 (0.001–0.431)	**0.043**
NLR	0.057 (−0.171 to 0.279)	0.617
PLR	0.013 (−0.214 to 0.238)	0.911
SII	0.075 (−0.153 to 0.296)	0.507
PNI	−0.132 (−0.348 to 0.097)	0.243
ALI	−0.113 (−0.330 to 0.116)	0.320
GPS	0.067 (−0.162 to 0.288)	0.556
CD8	0.062 (−0.165 to 0.284)	0.579
FOXP3	0.187 (−0.040 to 0.396)	0.095

Abbreviations: 95 % CI, 95 % confidence interval; LAT1, L-type amino acid transporter 1; ASCT2, alanine-serine-cysteine transporter 2; VEGFR2, vascular endothelial growth factor receptor 2; NLR, neutrophil to lymphocyte; PLR, platelet to lymphocyte; SII, systemic immune inflammation; PNI, prognostic nutritional index; ALI, advanced lung cancer inflammation; GPS, Glasgow prognostic score. Bold font indicates a statistically significant difference.

### 3.4 Survival analysis

**Fig. 2:**
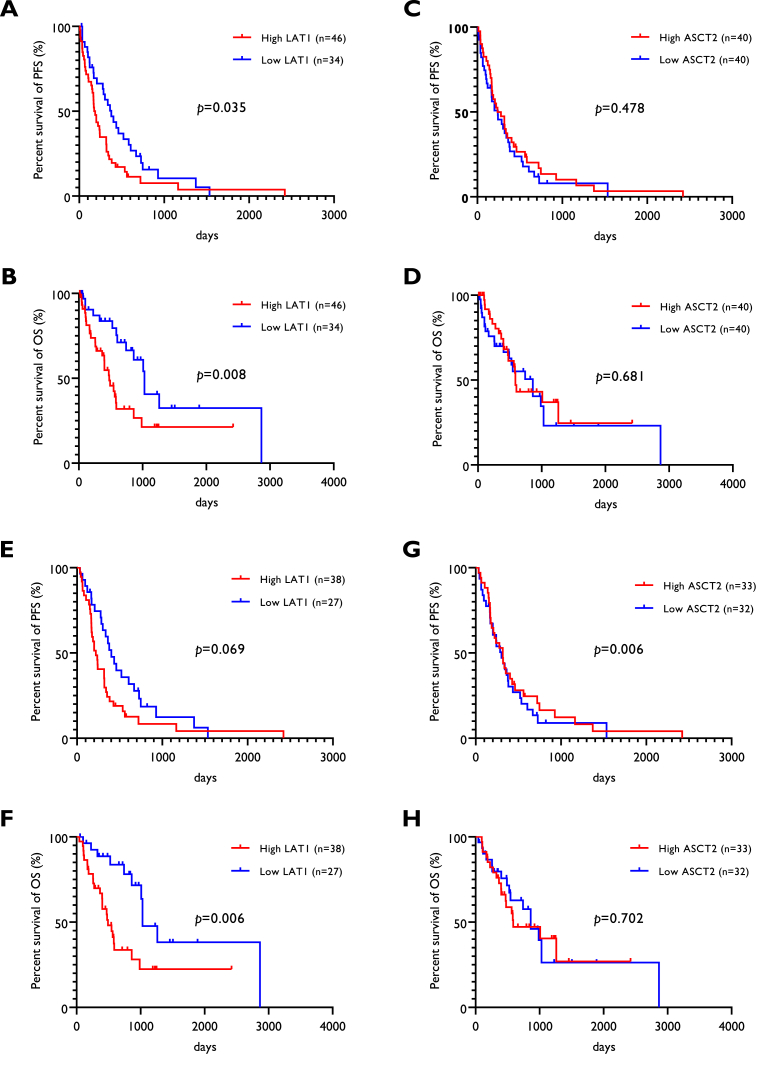
Kaplan–Meier curves of PFS and OS according to LAT1 and ASCT2 expression.High LAT1 expression was associated with a significantly shorter PFS (A) and OS (B) compared to low LAT1 expression in all patients (n = 80). However, no statistically significant difference in PFS (C) and OS (D) was observed between patients with high and low ASCT2 expression. Among the 65 patients receiving any systemic treatment, those with high LAT1 expression tended to have shorter PFS (E) than those with low LAT1 expression; however, a statistically significant difference in OS (F) was observed between patients with high and low LAT1 expression. There was no significant difference in PFS (G) or OS (H) according to the expression level of ASCT2.

The median PFS and OS of all patients were 237 and 603 days, respectively. Seventy-seven patients experienced recurrence after the initial treatment, and 45 died owing to progressive disease. Table 3 shows the univariate and multivariate survival analyses based on different variables for all patients (n = 80). The Kaplan–Meier survival curves of patients with high and low LAT1 (Fig. 2A, B, 2E, 2F) and ASCT2 (Fig. 2C, D, 2G, 2H) expression are shown in Fig. 2. According to the univariate log-rank test, performance status, histology, LAT1, PNI, ALI, and GPS were significant prognostic factors for PFS, while Ki-67, LAT1, NLR, SII, PNI, ALI, and GPS were significant predictors of OS (Table 3). Different variables with *p* < 0.05 were selected for subsequent multivariate analysis. Multivariate analysis confirmed that LAT1 expression was an independent prognostic factor for PFS and OS (Table 3). Next, the survival analysis focused on 65 patients who received systemic treatment with or without surgical resection (Table A1, online only). Univariate analysis identified PNI and GPS as significant factors for PFS and Ki-67, LAT1, SII, ALI, and GPS as significant factors for OS (Table A1). Multivariate analysis demonstrated that LAT1 was an independent prognostic factor for predicting poor PFS and OS.

**Table 3:** Univariate and multivariate survival analysis in all patients (n = 80).

Different variables		Progression-free survival					Overall survival				
		Univariate analysis		Multivariate analysis			Univariate analysis		Multivariate analysis		
		MST	*p*-value	HR	95 % CI	*p*-value	MST	*p*-value	HR	95 % CI	*p*-value
		(days)					(days)				
Age	<70/≥70 years	278/175	0.071				564/860	0.263			
Gender	Male/Female	237/317	0.275				603/759	0.928			
ECOG PS	0-1/2-3	320/161	**0.002**	1.498	0.755-2.974	0.248	740/326	0.145			
Smoking	Yes/No	243/198	0.541				740/603	0.669			
Asbestos	Yes/No	285/208	0.773				564/986	0.242			
Stage	1-2/3-4	208/278	0.063				860/587	0.121			
Histological type	Epi/Non-epi	303/155	**0.034**	1.701	0.962-3.008	0.068	740/546	0.411			
Ki-67 LI (%)	High/Low	208/303	0.068				476/1009	**0.009**	1.394	0.693-2.804	0.351
LAT1	High/Low	181/377	**0.035**	1.696	1.031-2.785	**0.037**	476/1029	**0.008**	2.973	1.466-6.029	**0.003**
ASCT2	High/Low	258/240	0.478				588/860	0.681			
VEGFR2	High/Low	318/187	0.210				860/581	0.181			
NLR	High/Low	170/318	0.069				489/986	**0.012**	0.759	0.270-2.131	0.600
PLR	High/Low	175/303	0.368				603/587	0.500			
SII	High/Low	187/318	0.140				489/1009	**0.002**	1.347	0.474-3.825	0.576
PNI	High/Low	362/169	**0.001**	0.579	0.294-1.140	0.114	986/489	**0.012**	0.716	0.346-1.480	0.367
ALI	High/Low	321/170	**0.018**	0.901	0.497-1.635	0.733	1009/469	**0.001**	0.520	0.149-1.814	0.305
GPS	0/1-2	352/175	**0.002**	0.640	0.348-1.177	0.151	1029/489	**0.001**	0.504	0.225-1.129	0.096
CD8	High/Low	285/171	0.758				588/850	0.972			
FOXP3	High/Low	221/243	0.805				603/740	0.994			

Abbreviations: LAT1, L-type amino acid transporter 1; ASCT2, alanine-serine-cysteine transporter 2; ECOG, eastern corporative oncology group; PS, performance status; LI, labeling index; epi/non-epi; epithelial type/non-epithelial type; VEGFR2, vascular endothelial growth factor receptor 2; NLR, neutrophil to lymphocyte; PLR, platelet to lymphocyte; SII, systemic immune inflammation; PNI, prognostic nutritional index; ALI, advanced lung cancer inflammation; GPS, Glasgow prognostic score; MST, median survival time; HR, hazard ratio; 95 % CI, 95 % confidence interval. Bold font indicates a statistically significant difference.

### 3.5 Relationship between inflammatory/nutritional induces and LAT1 expression

In all patients, high LAT1 expression was identified as a significant predictor of worse PFS in various subgroups, including those with stage III or IV disease, high PLR, high SII, low PNI, and high GPS. Additionally, subgroups characterized by stage III or IV disease, epithelial type, high PLR, high SII, and low ALI were closely associated with high LAT1 expression, predicting of poor OS (Table 4). Among the 65 patients who received systemic treatment, the association between LAT1 expression and inflammatory/nutritional indices was similar to that observed in the overall patient cohort (Table A2, online only).

**Table 4:** Relationship between inflammatory/nutritional indices and LAT1 expression.

Different variables		Progression-free survival		Overall survival	
		MST(days)	*p*-value	MST(days)	*p*-value
		LAT1 high/low		LAT1 high/low	
Stage	1–2	168/460	0.179	860/NR	0.598
	3–4	221/362	**0.028**	469/1029	**<0.001**
Histological type	Epi	229/403	0.130	489/1029	**0.010**
	Non-epi	110/336	0.099	258/NR	0.313
NLR	High	169/377	0.094	257/1029	0.092
	Low	317/362	0.331	587/1029	0.056
PLR	High	155/403	**0.0002**	402/1029	**0.009**
	Low	317/303	0.972	581/2866	0.129
SII	High	169/377	**0.012**	258/1029	**0.014**
	Low	317/362	0.487	986/1029	0.225
PNI	High	319/435	0.636	587/1029	0.063
	Low	130/291	**0.014**	399/1029	0.073
ALI	High	317/403	0.276	860/1029	0.123
	Low	109/278	0.069	257/740	**0.018**
GPS	High	168/336	**0.012**	599/839	0.299
	Low	317/478	0.674	986/1029	0.580

Abbreviations: LAT1, L-type amino acid transporter 1; epi/non-epi; epithelial type/non-epithelial type; NLR, neutrophil to lymphocyte; PLR, platelet to lymphocyte; SII, systemic immune inflammation; PNI, prognostic nutritional index; ALI, advanced lung cancer inflammation; GPS, Glasgow prognostic score; MST, median survival time; NR, not reached; Bold font indicates a statistically significant difference.

## 4 Discussion

In this clinicopathological study, we evaluated the prognostic significance of LAT1 expression in patients with PM. Our study successfully demonstrated that high LAT1 expression is an independent predictor of worse outcomes in patients with PM. LAT1 was highly expressed in 57.5 % of cases and closely correlated with tumor cell proliferation and glutamine transporters. We found that LAT1 is a crucial prognostic predictor of advanced-stage disease, high inflammation, and low nutritional status. However, in the present study, the therapeutic strategies, including chemotherapy followed by surgery, chemotherapy alone, immunotherapy, or best supportive care, were heterogeneous, which may have introduced bias into our results. Further studies are warranted to elucidate the prognostic role of LAT1 expression in patients receiving homogeneous therapeutic strategies.

Our study is a follow-up of the previous study [8]. In comparison with previous small study, however, the results of the current study confirmed the novelty that overexpression of LAT1 in addition to high inflammatory and low nutritional status strongly encourages worse outcome for the patients with PM. Besides, sufficient sample size is essential for the prognostic prediction of LAT1 expression. Thus, we believe that the present study is helpful for the prognostic significance of LAT1 expression in the patients with PM, as an independent cohort from previous small study [8].

A previous meta-analysis demonstrated that LAT1 is highly expressed in many human neoplasms and depicted a close relationship between its expression and worse outcomes after treatment [5]. Recently, Okano et al. reported the first-in-human phase I study of nanvuranlat (JPH203) as an LAT1 inhibitor in advanced solid tumors [20]. This study confirmed the safety and efficacy of LAT1 inhibitors in the treatment of human cancer [20]. Furthermore, a recent randomized phase II study reported that the therapeutic response of nanvuranlat as an LAT1 inhibitor was significantly superior to that of the placebo control in patients with previously treated biliary tract cancer and met the primary endpoint of this study [21]. Therefore, the therapeutic efficacy of LAT1 inhibitors has been clinically proven for the treatment of a limited number of neoplasms. As clinical trials of LAT1 inhibitors have not yet been conducted in other human cancers, further investigation is warranted to plan clinical studies of LAT1 inhibitors in thoracic neoplasms, including PM.

The expression rates of LAT1 vary across different types of cancers, as follows: 53 % in pancreatic cancer, 43 % in breast cancer, 60 % in non-small cell lung cancer, and 80 % in colorectal cancer [16]. In our study, patients with PM exhibited a positive LAT1 expression rate of 57.5 %. This suggests that the expression of LAT1 in PM is similar to that observed in other solid tumors. Overexpression of LAT1 is closely associated with tumor cell proliferation and poor outcomes in patients with lung cancer [4]. The clinical significance of LAT1 expression may play a crucial role in tumor pathogenesis and survival in thoracic neoplasms, including PM. In addition, our study indicated that LAT1 expression was not associated with the tumor immune microenvironment, as determined by TILs in patients, in patients with PM. A previous report suggested that LAT1 increases the activation of the mammalian target of rapamycin (mTOR) pathway [22]. The mTOR pathway, in turn, suppresses Treg cells and promotes the differentiation of CD8-positive T cells [23,24]. Increased LAT1 expression in breast cancer has been reported to be closely correlated with increased TIL levels [25]. The association between LAT1 expression and TILs may differ according to individual neoplasms.

In the current study, univariate analysis revealed that nutritional markers such as PNI, ALI, and GPS were significant predictors. Although LAT1 is well known as a marker of nutrient uptake, low nutritional indices are predictive of worse outcomes following any treatment in patients with PM. Interestingly, in a highly inflammatory and low nutritional environment, LAT1 expression was more predictive of outcomes for patients with PM. A recent experimental study reported that LAT1 plays a crucial role in the activation of T cell subsets under inflammatory conditions, suggesting a new therapeutic target for rheumatoid arthritis [26]. However, the pathogenic significance of LAT1 under inflammatory conditions in tumor specimens remains unclear. Further investigation is warranted to elucidate the function of LAT1 in tumor cells under highly inflammatory or low nutritional conditions.

The present study has several limitations. First, our enrolled patients received heterogeneous treatment because of the long registration period. Further clinicopathological studies of patients treated with a uniform regimen in a first-line setting are necessary. Second, the prognostic significance of LAT1 expression according to individual regimens remains unclear. Chemotherapeutic regimens of cisplatin plus pemetrexed, cisplatin plus gemcitabine, nivolumab, or ipilimumab plus nivolumab were chosen based on the judgement of the chief physicians. A recent study demonstrated that the efficacy of ipilimumab plus nivolumab is significantly superior to that of platinum-based chemotherapy [6]. Further studies should focus on immunotherapy as the first-line treatment to evaluate the clinical significance of LAT1 expression. Finally, little is known about the detailed mechanism underlying tumor shrinkage induced by the inhibition of LAT1 in mesothelioma cells. Many tumor cell lines have been investigated for the inhibition of LAT1, and evidence of tumor reduction has been provided by experimental studies [1,2]. In addition, we defined a cut-off value for Ki-67, CD8, FOXP3 and ASCT2 as median value based on the evidence of previous studies [18,19]. Nowadays, there is not established definition to decide the cut-off value of these markers. The definition of cut-off value is different according to individual studies. However, our cut-off value may introduce bias because of limited population.

In conclusion, LAT1 was highly expressed in patients with PM, and was identified as an independent predictor of worse outcomes following any treatment. LAT1 was more predictive of worse outcomes in high-inflammatory and low-nutritional environments. Although the development of any molecular targeting agents is expecting for the patients with PM, a considerable experimental study is essential for the next step to clinical trial. Recently, a randomized phase II study of the efficacy of nanvuranlat as LAT1 inhibitor was reported for the patients with previously treated advanced biliary tract cancers, indicating that nanvuranlat significantly improved PFS compared to placebo group [27]. However, it is difficult to speculate the potential of LAT1 inhibitor to the patients with PM based on the results of our current study. Further study is warranted to elucidate the biological characteristics of PM from the viewpoint of amino acid metabolism.

Further studies are warranted to investigate the clinical efficacy of LAT1 inhibitors in patients with advanced PM.

## Funding

Our study did not receive any research funding from the commercial or public sectors.

## Data availability

All data generated or analyzed during this study are included in this article.

## Ethical approval

All procedures involving human participants performed in this study were in accordance with the ethical standards of the institutional and/or national research committees and the 1964 Declaration of Helsinki and its later amendments or comparable ethical standards.

## Consent for publication

Not applicable.

## CRediT authorship contribution statement

**Ryo Taguchi:** Writing – review & editing, Writing – original draft, Methodology, Investigation, Formal analysis, Data curation, Conceptualization. **Kyoichi Kaira:** Writing – review & editing, Writing – original draft, Methodology, Investigation, Formal analysis, Data curation, Conceptualization. **Yu Miura:** Data curation. **Tetsuya Umesaki:** Data curation. **Atsuto Mouri:** Data curation. **Hisao Imai:** Data curation. **Hiroshi Kagamu:** Data curation. **Masanori Yasuda:** Data curation. **Yoshikatsu Kanai:** Writing – review & editing, Writing – original draft, Methodology, Conceptualization. **Hiroyuki Nitanda:** Data curation. **Hironori Ishida:** Data curation. **Hirozo Sakaguchi:** Data curation.

## Declaration of competing interest

The authors declare that they have no known competing financial interests or personal relationships that could have appeared to influence the work reported in this paper.
