# Supplementary material for: Prognostic significance of LAT1 expression in pleural mesothelioma
Source: Heliyon. 2024 Sep 3;10(17):e37414. doi: 10.1016/j.heliyon.2024.e37414 (PMC11407025; doi:10.1016/j.heliyon.2024.e37414)
Supplement: Multimedia component 1 [file mmc1.docx]

**Table A2. Relationship between inflammatory/nutritional indices and LAT1 expression (n=65)**

| Different variables | | Progression-free survival | | Overall survival | |
| --- | --- | --- | --- | --- | --- |
|  |  | MST(days)  LAT1 high/low | *p*-value | MST(days)  LAT1 high/low | *p*-value |
| Stage | 1-2 | 545 / 621 | 0.463 | 622 / 598 | 0.667 |
|  | 3-4 | 256 / 456 | **0.013** | 460 / 1575 | **<0.0001** |
| Histological type | Epi | 442 / 805 | 0.457 | 571 / 1534 | **0.016** |
|  | Non-epi | 261 / 426 | 0.108 | 786 / 700 | 0.527 |
| NLR | High | 306 / 512 | 0.128 | 352 / 984 | **0.049** |
|  | Low | 539 / 539 | 0.320 | 653 / 1738 | 0.067 |
| PLR | High | 183 / 605 | **0.002** | 353 / 972 | **0.008** |
|  | Low | 522 / 478 | 0.988 | 635 / 1890 | 0.119 |
| SII | High | 248 / 521 | **0.024** | 384 / 939 | **0.005** |
|  | Low | 544 / 550 | 0.510 | 723 / 1799 | 0.319 |
| PNI | High | 563 / 593 | 0.596 | 646 / 894 | 0.087 |
|  | Low | 195 / 390 | **0.034** | 366 / 1407 | **0.045** |
| ALI | High | 481 / 575 | 0.197 | 700 / 1828 | 0.117 |
|  | Low | 288 / 471 | 0.167 | 343 / 908 | **0.012** |
| GPS | High | 211 / 430 | 0.071 | 371 / 1589 | **0.002** |
|  | Low | 568 / 592 | 0.493 | 747 / 1022 | 0.455 |

Abbreviations: LAT1, L-type amino acid transporter 1; epi / non-epi; epithelial type / non-epithelial type; NLR, neutrophil to lymphocyte; PLR, platelet to lymphocyte; SII, systemic immune inflammation; PNI, prognostic nutritional index; ALI, advanced lung cancer inflammation; GPS, Glasgow prognostic score; MST, median survival time; NR, not reached; Bold font indicates a statistically significant difference.
